# Statistical Associations between Vestibular Pathologies and Hypothyroidism: A Retrospective Study

**DOI:** 10.3390/jcm13041099

**Published:** 2024-02-15

**Authors:** Virginie Bougerolle, Rhizlane El Khiati, Abdessadek El Ahmadi, Brahim Tighilet, Stéphane Besnard, Christian Chabbert

**Affiliations:** 1 Practice of a Physiotherapy and Vestibular Rehabilitation, 59140 Dunkerque, France; bougerolle.virginie@orange.fr; 2 Research Centre in Psychology and Neurosciences, Aix Marseille University-CNRS, UMR7077, Team VESTIMED, 13331 Marseille, France; ghizlaineelkhiati@gmail.com (R.E.K.); abdessadek.el-ahmadi@univ-amu.fr (A.E.A.); brahim.tighilet@univ-amu.fr (B.T.); 3 Research Group on Vestibular Pathophysiology, Unit GDR2074 CNRS, 13331 Marseille, France; stephane.besnard@unicaen.fr; 4 UR VERTEX 7480, Université de Caen-Normandie, 14032 Caen, France

**Keywords:** neurotology, vertigo, unsteadiness, hormones, hypothyroidism

## Abstract

The association between vestibular pathologies and thyroid hormone disorders has been known for several decades. However, very little information is available on the types of vestibular symptoms that may be affected by altered thyroid hormone levels. The aim of this study was to provide patient data in order to identify statistical associations between vestibular pathologies and thyroid hormone disorders. A retrospective review of the records of 422 patients seen for physiotherapy treatment of vertigo was carried out. Statistical analysis of the data was performed using logistic regression, providing Chi2 and Odds Ratio statistics. Our results show that hypothyroidism statistically significantly increases the expression of certain symptoms, such as vestibular instability and gait disorders, in vestibular pathologies such as Menière’s disease or central vertigo. By analyzing patient data, our study provides new evidence of dependence between altered thyroid status and the expression of vestibular pathologies.

## 1. Introduction

Several studies published over nearly six decades have established a correlation, or more broadly a dependency, between thyroid disorders and the onset of vertigo [1,2]. The first study associated with thyroid hormone disorders with vestibular disorders dates back to 1959. Shambaugh demonstrated a relationship between hypothyroidism and Menière’s disease [3]. This finding was later confirmed in the 1970s by Powers [4], who found clinically significant hypothyroidism in 17% of his 98 patients with Menière’s disease. The discovery and description of Pendred syndrome, a specific genetic disorder affecting both inner ear development and thyroid function, helped to confirm this link between endocrine and vestibular functions [5,6].

### 1.1. Hypothyroidism and BPPV

In 2017, Chiarella et al. [7] published an article reviewing the various existing publications on the relationship between vestibular dysfunction and Hashimoto’s thyroiditis. They referred to the study by Modugno in 2010 [8], which counted 34/70 BPPV (Benign paroxysmal positional vertigo) patients with high levels of anti-thyroid antibodies. In 2009, Papi had already noted elevated levels of anti-thyroid peroxidase (anti-TPO), anti-thyroglobulin, and thyroid-stimulating hormone receptor antibodies in a group with vestibular disorders compared with the healthy group [9]. The following year, he found 18% of patients with signs of BPPV in the group of patients with Hashimoto’s thyroiditis compared with 2% in the control group. As these 18% were all euthyroid, he assumed a mechanical stimulation of immune complexes and the coexistence of an inner ear microangiote in the context of a multi-visceral disease at the origin of the link between metabolic disorders and vestibular disorders [10]. Conversely, in a 2015 study, Sari [11] found no difference in the level of anti-thyroid antibodies between the BPPV group and the control groups. In 2021, Teggi and colleagues [12] published a study on the role of vascular disorders, migraines, and anti-thyroid antibodies in patients with recurrent BPPV. They found a higher level of antibodies in the female population, which is already more prone to BPPV and its recurrences. They stated that the presence of antibodies is the main causal factor in the recurrence of BPPV when it occurs between the ages of 40 and 60. They suggested a possible overlap between autoimmune diseases and BPPV, either through a cross-reaction mechanism (the inner ear may share common antigens with certain organisms such as viruses or bacteria) or, like Papi, mechanical stimulation by immune complexes in the context of a multi-visceral autoimmune disease. In the same year, Choi, Kim et al. [13] carried out the first large study with a control group on the rate of various thyroid diseases in BPPV patients. The result was that hypothyroidism was linked to BPPV in all women and men over the age of 65. As in Papi and Teggy’s studies, they assert that the state of thyroid function is of little importance in the risk of BPPV, unlike hormonal status (the level of antibodies is more important in assessing the risk of BPPV than the level of thyroid hormones themselves). The origin of this link between hypothyroidism and BPPV suggests an autoimmune dysfunction or a disturbance in endolymphatic flow or insufficient blood flow in the inner ear. They highlighted the complexity and uncertainty that still exists today about this relationship between hypothyroidism and vestibular disorders. Above all, the high level of anti-thyroid antibodies certainly plays a major role, and consequently, the onset and recurrence of BPPV are probably of autoimmune origin.

### 1.2. Hypothyroidism and Meniere Disease

In 1995, Rybak [1] reviewed the various studies already carried out on the effect of different metabolic disorders on vestibular function. With regard to hypothyroidism, he referred to the article by Powers [4], who had found 17% hypothyroidism in 98 Menière patients. However, treatment with thyroid hormones only controlled symptoms in 3 out of 17 patients. In 2007, Fattori et al. [14] evaluated the association between thyroid autoimmunity and Menière’s disease and found that 38% of Menière’s patients had significant levels of anti-thyroid antibodies (mainly anti-TPO antibodies) compared with 7 and 12% in the control groups. They concluded that autoimmunity may play a pathogenic role in the development of the disease. In 2012, Santos and Bittar [15] studied the prevalence of carbohydrate, lipid, and thyroid disorders in vestibular disorders and confirmed a higher prevalence of hypothyroidism in the Menière group (13.6%) than in the control group (10%), thus reinforcing the hypothesis of the influence of metabolic disorders on the etiology of vestibular symptoms. In 2014, Chiarella et al. [16] studied the relationship between vestibular lesions and Hashimoto’s thyroiditis, finding a high prevalence of impaired vestibular function in Hashimoto’s patients with elevated anti-TPO antibody levels, considered a risk factor for the development of vestibular dysfunction and thus confirming the predominant role of autoimmunity. In 2016, Santosh and Rao [17] investigated the incidence of hypothyroidism in Menière’s disease and the effect of hypothyroidism treatment on disease symptoms. They found that the incidence of hypothyroidism is high in Menière’s patients and that all patients who received treatment for hypothyroidism had a significant improvement in their symptoms. In 2019, Lin et al. [18] published a study on the correlation between hypothyroidism and Menière’s disease and again found a high prevalence of Menière’s disease in hypothyroid patients, particularly in those over 50 years of age, with a significant time interval of less than 5 years for the onset of the disease.

Hypothyroidism without treatment is associated with a high risk of Menière’s disease, and this risk decreases after treatment without the difference being significant, so the effect of L-Thyroxine treatment remains controversial. Finally, in 2020, Kim et al. [19] conducted a study on the relationship between thyroid disease and Menière’s disease and found a higher rate of thyroid disease and a higher rate of L-Thyroxine intake in the Menière’s group compared with the control group. Hypothyroidism is associated with Menière’s disease, particularly in women under 65. They suggested that thyroid dysfunction leads to inflammatory and metabolic changes and has an impact on inflammation of the inner ear and homeostasis of endolymphatic flow, thus causing hydrops. They also returned to the notion of an abnormal autoimmune response. In conclusion, we can see from all these publications that the link between hypothyroidism and dizziness has been known for many years and has been confirmed by various studies, even though some authors contradict it. However, the mechanism of origin remains unknown so far.

### 1.3. Impact of Hypothyroidism on Vestibular Disorders Symptoms

Another important issue concerns the types of vestibular symptoms that may be affected under hypothyroidism conditions and further treatment with thyroxine. The 2016 study from Santosh and colleagues carried out on patients suffering from Menière’s disease demonstrated that thyroid hormone levels were likely to modulate the expression of sensation of rotation, tinnitus, hearing loss, and fullness of the ear [17] and that these symptoms were significantly reduced in hypothyroid patients treated orally with thyroxine. This observation suggested that other types of vestibular symptoms could also potentially be influenced by thyroid hormone levels and that pharmacological action to modulate thyroid hormone blood levels could be part of the therapeutic arsenal to reduce them. However, to date, there is no precise information on the different types of vestibular symptoms that may be affected by hypothyroidism. To directly address this question, we studied a population of 422 patients attending a vestibular rehabilitation clinic, stratified into six groups of vestibular pathologies (BPPV, unilateral vestibular deficit, Menière’s disease, kinetosis, chronic instability, and central vertigo). In these patients, we analyzed the expression of several symptoms classically encountered in these vestibular pathologies: rotatory vertigo, instability, gait disorders, and visual dependence. The level of expression of these different symptoms was compared between euthyroid (no thyroid dysfunction) and hypothyroid patient populations, as well as between male and female populations. Statistical analysis of patient data shows that hypothyroidism statistically significantly increases the expression of specific symptoms in conditions such as Menière’s disease and central vertigo. While most of the results of our study corroborate the relationship between symptoms and vestibular pathologies, others, less expected, reveal a direct impact of thyroid hormone alterations on the expression of vestibular disorders.

## 2. Materials and Methods

### 2.1. Collection of Bibliographical Data

**Figure 1:**
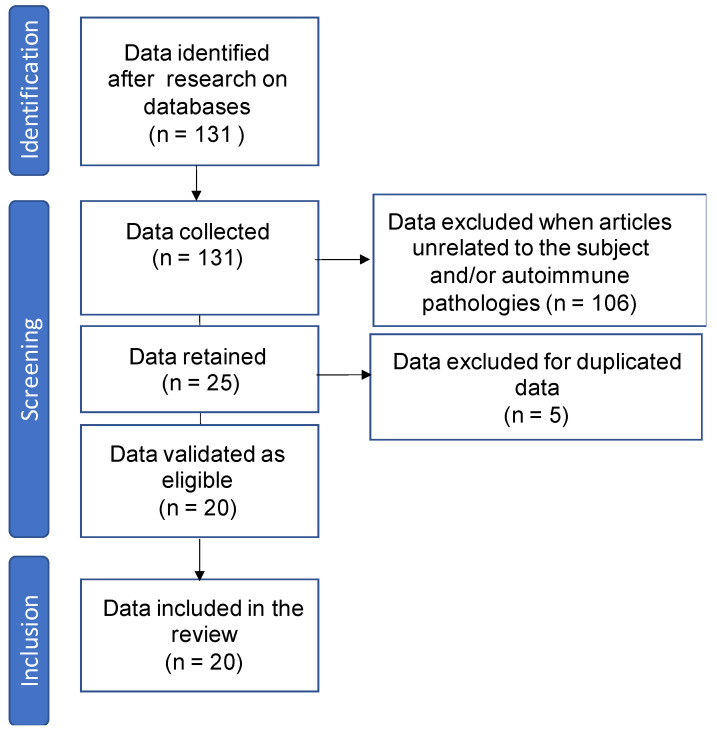
Flowchart presenting the steps of the data collection process.

We carried out a bibliographic study on the PubMed platform to identify publications over the last ten years dealing with the link between thyroid hormones and vertigo. The search was carried out using a combination of the following keywords: Dizziness and hormones, Dizziness and T3 (Triiodothyronine), Dizziness and T4 (thyroxine), Dizziness and hypothyroidism, Dizziness and thyroid, Meniere and thyroid, Meniere and T3, Meniere and T4, Meniere and thyroid hormones, Meniere and hypothyroidism, BPPV and thyroid, BPPV and T3, BPPV and T4, BPPV and thyroid hormones, BPPV and hypothyroidism, Vestibular migraine and thyroid, Vestibular migraine and T3, Vestibular migraine and T4, Vestibular migraine and thyroid hormones, Vestibular migraine and hypothyroidism. A total of 131 articles were initially identified. After excluding duplicates, thyroid autoimmune diseases, and articles unrelated to the subject, 20 articles were retained [2,17,18,19,20,21,22,23,24,25,26,27,28,29,30,31,32], providing access to the full text. The stages in the data collection process are described in Figure 1.

### 2.2. Participants

A retrospective review of the records of patients seen in consultation as part of a prescription for physiotherapy management of vertigo between September 2019 and October 2021 was carried out. A total of 422 patients diagnosed with vestibular disorders were included in this study. Patients consulting for vertigo but without a precise diagnosis were excluded from this study. This study was validated by the Health Research Ethics Committee of the Centre Hospitalier Universitaire Caen Normandie (ID number 549823). Patients were informed that their data would be used anonymously. Patients were referred to the physiotherapy practice by their general practitioner or ear and nose throat specialists. The vestibular pathology was assessed on the basis of previous medical diagnosis, an interview, and a physiotherapy assessment, including examination under videonystagmoscopy (search of spontaneous nystagmus, presence or absence of gaze nystagmus, search for BPPV using provocative maneuvers, the study of the VVOR (visuo vestibulo ocular reflex), checking IFO (ocular fixation index), study of the VOR (vestibulo–ocular reflex), HIT (Head Impulse test) search for catch-up saccades, HST (Head shaking test). Six pathologies were selected: BPPV, unilateral deficit, Menière’s disease, kinetosis, chronic instability, and central vertigo (vertigo of neurological origin). BPPV patients attended the vestibular rehabilitation program when the maneuvers could not be achieved by the referring physician. Patients were included in the central vertigo group on the basis of a medical prescription indicating a central lesion or a nystagmus study showing signs of centrality. The thyroid status of patients was established by referring to the subjects’ medical records from a thyroid specialist.

### 2.3. Data Analysis

We first studied the relationships between symptoms and vestibular pathologies, then the relationships between symptoms and vestibular pathologies and the different thyroid states, i.e., hypothyroidism and euthyroidism (control group). We used logistic regressions and contingency tables crossing qualitative variables. Chi2-type tests were calculated, in particular, to test the independence of two qualitative variables. A *p*-value < 0.05 means that there is a significant association or dependence between two variables. We then used the Odds Ratio statistic to measure the degree of dependence between variables. An Odds Ratio of less than 1 means that an event is less frequent in one group than in the other. An Odds ratio = 1 means that an event is equally present in both groups. An Odds ratio greater than 1 means that an event is more frequent in one group than in the other.

## 3. Results

### 3.1. Demographics and Clinical Characteristics of the Study Group

A total of 422 patients were included in the present study. Demographic data and clinical characteristics of the study group are presented in Table 1. The study group comprised 27.5% men and 72.5% women with a mean age of 61.5 ± 14.7 years (60.4 ± 15.4 years for men, 61.9 ± 14.5 years for women) and age ranges of 19 to 88 years for men and 24 to 91 years for women.

#### 3.1.1. Pathologies

**Figure 2:**
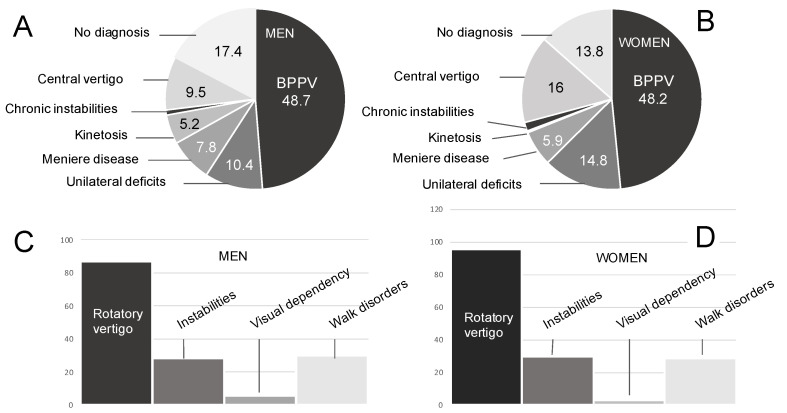
Repartition of pathologies and symptoms. Repartition of pathologies in men (**A**) and women (**B**). Repartition of symptoms in men (**C**) and women (**D**).

The vestibular pathologies were distributed as follows between the two groups (Figure 2A,B). In the male group, 48.7% had BPPV, 10.4% unilateral deficit, 7.8% Meniere’s disease, 5.2% kinetosis, 0.9% chronic instability and 9.5% central vertigo. In total, 17.4% of male patients had no precise diagnosis. In the female group, 48.2% had BPPV, 14.8% unilateral deficit, 5.9% Meniere’s disease, 0.3% kinetosis, 1% chronic instability and 16% central vertigo. In total, 13.8% of female patients had no precise diagnosis.

#### 3.1.2. Expressed Symptoms

The symptoms expressed by the patients were divided into 87.1% rotatory vertigo, 28.4% instability, 6% visual dependence, and 30.2% walking difficulties in the men, and 91.5% rotatory vertigo, 29.7% instability, 2.9% visual dependence and 28.7% walking difficulties in the women (Figure 2C,D).

#### 3.1.3. Thyroid Status

Thyroid status was as follows: 9.5% of men and 15.3% of women were hypothyroid, and all were treated with synthetic thyroid hormones.

### 3.2. Studies of Statistical Associations between Vestibular Pathologies and Expressed Symptoms

The results of the search for associations between vestibular pathologies and symptoms are presented in Table 2. In the total patient population, there was a statistically significant relationship of dependence between BPPV and, in ascending order, rotatory vertigo (*p* < 0.001 and OR = 10.549), gait disorders (*p* < 0.001 and OR = 0.222), instability (*p* < 0.001 and OR = 0.133) and visual dependence (*p* < 0.001 and OR = 0.029). Patients with BPPV were statistically significantly more likely to present, in ascending order, these symptoms than patients with other vestibular pathologies. There is a significant relationship of dependence between unilateral deficit and, in ascending order, gait disorders (*p* < 0.001 and OR = 3.387) and instability (*p* < 0.001 and OR = 3.064). Patients with a unilateral deficit were statistically significantly more likely to present, in ascending order, these symptoms than patients with other vestibular pathologies. There was a significant relationship between Menière’s disease and, in ascending order, instability (*p* < 0.001 and OR = 5.211) and gait disorders (*p* = 0.002 and OR = 3.290). Patients with Menière’s disease had a statistically highly significant greater risk of presenting, in ascending order, these symptoms than patients with other vestibular pathologies. There was a significant relationship between kinetosis and, in ascending order, visual dependence (*p* < 0.001 and OR = 91.818) and rotatory vertigo (*p* < 0.001 and OR = 0.015). Patients with kinetosis were statistically significantly more likely to present these symptoms in ascending order than patients with other vestibular pathologies. There was a significant relationship between central vertigo and, in ascending order, instability (*p* < 0.001 and OR = 3.354), gait disorders (*p* = 0.004 and OR = 2.267), and rotatory vertigo (*p* < 0.001 and OR = 0.180). Patients with central vertigo were statistically significantly more likely to present these symptoms in ascending order than patients with other vestibular pathologies. There was a significant relationship between chronic instability and rotatory vertigo (*p* = 0.006). Patients with chronic instability were statistically significantly more likely to suffer from rotatory vertigo than patients with other vestibular pathologies (OR = 0.101).

The study of the gender effect on the results of the search for associations between vestibular pathologies and symptoms shows that men present more rotatory vertigo than women in BPPV (OR = 34.495 vs. OR = 6.079). Men also had significantly more instability than women in conditions such as Menière’s disease (OR = 10,904 vs. OR = 3930) and central vertigo (OR = 5317 vs. OR = 3015). As for women, they have significantly more visual dependence than men when it comes to kinetosis (OR = 105 vs. OR = 71.333).

### 3.3. Studies of Statistical Associations between Clinical Characteristics and Thyroid Conditions

Separate analysis of the euthyroid and hypothyroid patient populations reveals specific associations between clinical features and thyroid conditions. The results are presented in Table 3. When considering only the euthyroid population, we find the same significant relationships and ORs reported above between vestibular disease and the symptoms considered for patients with BPPV, unilateral deficit, kinetosis, and central vertigo. On the other hand, euthyroid patients with Menière’s disease were statistically significantly more likely to present, in ascending order, instability (*p* < 0.001 and OR = 4.757), visual dependence (*p* = 0.022 and OR = 4.312) and gait disorders (*p* = 0.016 and OR = 2.688), than patients with other vestibular diseases. Similarly, there was a significant relationship between chronic instability and, in ascending order, gait disorders (*p* < 0.001 and OR = 28.731), instability (*p* = 0.010 and OR = 10.480), and rotatory vertigo (*p* < 0.001 and OR = 0.074). Euthyroid patients with chronic instability were statistically significantly more likely to present these symptoms in ascending order than patients with other vestibular pathologies (OR = 0.101).

If the hypothyroid population is considered in isolation, hypothyroidism significantly increases the relationship between Menière’s disease and instability (*p* = 0.028 and OR = 9.067) and Menière’s disease and gait disorders (*p* = 0.020 and OR = 10). Hypothyroidism also increased the significant relationship between central vertigo and instability (*p* = 0.003 and OR = 9.625) and between central vertigo and gait disorders (*p* = 0.020 and OR = 5.500).

The study of the gender effect on the associations mentioned above shows that if we consider the population of women or men separately within the population of hypothyroid patients, the association between Menière’s disease and gait instability or walking disorders is no longer significant in either men or women. The association between central vertigo and walking disorders is no longer significant in hypothyroid men, whereas it remains so in the population of women taken separately (*p* = 0.019 and OR = 6.750). The effect remains highly significant (*p* = 0.004 and OR = 11.813) in hypothyroid women with regard to central vertigo and unsteadiness, whereas it is less systematic in hypothyroid men (*p* = 0.012 and OR = 5.317).

## 4. Discussion

The demonstration of relationships of dependence between the four symptoms and the different vestibular pathologies retained in the present study is not surprising. These associations were previously reported by other authors and included in the diagnostic criteria for each of the vestibular pathologies published by the Classification Committee of the Barany Society and the American Academy of Otolaryngology-Head and Neck Surgery (AAO-HNS) in charge of establishing a standardized international diagnosis of the different types of vestibular pathologies, or in other reference reviews. In our sample of patients, we found a high preponderance of gait disorders and instability in BPPV patients, patients with a unilateral deficit, and Menière patients, as previously reported [11,12,32,33,34]. We also found a preponderance of rotatory vertigo in patients with kinetosis, central vertigo, or chronic instability, as previously reported [35,36,37].

On the other hand, our observations show that in a situation of thyroid dysfunction, instability, and gait disorders are increased in Menière’s disease or central vertigo. To the best of our knowledge, this observation has never been reported before by other authors. However, in 2022, Hu et al. [23] suggested that hypothyroidism is a risk factor for Menière’s disease. Likewise, Tricarico et al. [27] speculated that patients with hypothyroidism have an increased risk of recurrent BPPV, which is particularly high in hypothyroid patients with positive thyroid antibodies, suggesting an association between autoimmunity and recurrent vertigo. In addition, our observation is similar to the study by Hsu et al. [38], who demonstrated a relationship between hypothyroidism and tinnitus, another symptom encountered in vestibular pathologies such as Menière’s disease. These authors found that patients with hypothyroidism displayed an increased risk of developing tinnitus.

A recent study by Rastoldo et al. [28] carried out in a rat model of unilateral vestibular loss (unilateral transection of the vestibular nerve) showed that early administration of L-Thyroxine significantly affected behavioral manifestations of vestibular imbalance. Circling, rearing, head tilt, and retropulsion, several functional signs observed within the first days following a unilateral vestibular loss, were especially decreased in rat groups treated with L-Thyroxine compared to animals administrated with saline solution. Thyroxine treatment also significantly improved both the gait and displacement of the animals. Altogether, these observations confirmed the benefits of thyroid hormones on the symptoms in situations of unilateral vestibular loss. A tentative interpretation of the cellular mechanisms behind this effect is proposed in this study. Interestingly, it has to be noted that raising thyroid hormone levels, even in euthyroid conditions, could reduce certain symptoms of acute peripheral vestibulopathy. The prospect of being able to test the effects of T4 administration in hypothyroid rats could confirm the results observed in patients of the present study.

The observations made in the present study can potentially be explained by the fact that a thyroid hormone deficiency can have an impact on various biological functions, such as basic cell metabolism or muscle energy control. Such a condition could actually exacerbate the symptoms, such as instability, encountered in the pathologies concerned. This observation also applies to gait disorders, which, as with the unsteadiness detailed above, could be exacerbated in vestibular patients with hypothyroidism.

The question that remains from these observations, however, is the fact that the patients considered to be hypothyroid in our study are all being treated with daily administration of thyroxine. We might legitimately have expected this treatment, which aims to restore thyroid hormone blood levels, to erase or reduce the differences in the expression of vertigo symptoms between hypothyroid and euthyroid patients. The thyroid hormones are, in fact, well known to have an overall stimulating effect on metabolism in most of the body’s tissues. By speeding up the basic metabolism, they increase the body’s consumption of energy and oxygen. They increase protein biosynthesis activity and accelerate glycogen breakdown and glucose biosynthesis through gluconeogenesis. Thyroid hormones also stimulate cholesterol breakdown and increase the number of LDL receptors, which accelerates lipolysis. In the brain, they stimulate the production of myelin, neurotransmitters, and axon growth. This set of tissue effects provides a general stimulation of the body’s functions, which should help it to cope better with the postural and motor alterations resulting from vestibular pathologies.

Specifically in the vestibular field, thyroid hormones exert a direct action on the neuronal circuits involved in the vestibular compensation process [28]. This study demonstrated, for the first time, the presence of both types of thyroid hormone receptors (TRα and TRβ) and the iodothyronine type II deiodinase enzyme (DIO2) in the vestibular nuclei (VN), attesting to a local action of L-T4. The results of this study also confirm the metabolic effects of L-T4 treatment by reporting an increase in the number of cytochrome oxidase-labeled neurons in the VN three days after injury. In Addition, L-T4 treatment modulates the glial response by reducing the number of microglia and oligodendrocytes in the brainstem vestibular secondary neurons three days after unilateral vestibular neurectomy and increases cell proliferation. The survival of newly generated cells in deafferented vestibular nuclei is not affected, but microglial rather than neuronal differentiation is enhanced by L-T4 treatment. Microglia, known to play a key role in various adaptive plasticity processes (neurogenesis, modulation of neuronal excitability, etc.), is a prime target for L-T4. Here again, these effects, if applied to humans, should make it possible to cope better with the symptoms encountered in vestibular pathologies. However, it appears from the present study that the daily administration of thyroxine, although intended to restore normal blood levels of thyroid hormones, is not sufficient to provide the expected benefits on the symptoms of the vestibular pathologies mentioned above. Further studies should be undertaken to explore the complex action of thyroxine on the vestibular system and its modulating effects on the symptoms of vestibular disorders.

The question of the gender effect on the associations revealed in our study is an interesting issue, as in certain cases, such as the association between Menière’s disease and gait instability or disorders, the separate analysis of men and women results in the loss of the statistical significance found in the overall group. On the other hand, in other cases, such as the association between central vertigo and gait disorders or central vertigo and instability, the female population shows a high level of statistical significance compared with the male population, demonstrating that they are more specifically affected by these associations.

### Limitations of the Study

We must take into account the fact that our sample is special. It is, in fact, a sample of patients seen at the vestibular rehabilitation practice, the vast majority of whom consult for BPPV. As a result, for certain categories of patients who are under-represented in terms of numbers (i.e., hypothyroid patients presenting with kinetosis hypothyroid men), we are unable to demonstrate significant relationships. This study should, therefore, be completed by a study including a larger sample of patients and, in particular, equivalent samples of hypothyroid, hyperthyroid, or euthyroid patients.

## 5. Conclusions

Our results show that hypothyroidism statistically significantly increases the expression of certain symptoms, such as vestibular instability and gait disorders, in vestibular pathologies such as Menière’s disease or central vertigo. By analyzing patient data, our study provides new evidence of dependence between altered thyroid status and the expression of vestibular pathologies.

## Figures and Tables

**Table 1:** Demographics and clinical characteristics of the study group.

Variable	Mean ± SD	Range
Gender n (%)		
Women	306 (72.5)	
Men	116 (27.5)	
Age (years)	61.5 ± 14.7	
Women	61.9 ± 14.5	24–91
Men	60.4 ± 15.4	19–88

**Table 2:** Logistic regression analysis is used for binary outcome data (e.g., Rotatory Vertigos) to model associations with each predictor (e.g., BPPV), represented in terms of odds ratios (OR).

Symptoms/ Pathologies	Rotatory Vertigo	Instabilities	Visual Dependency	Walk Disorders
BPPV	OR = 10.549 *p* < 0.001	OR = 0.133 *p* < 0.001	OR = 0.029 *p* < 0.001	OR = 0.222 *p* < 0.001
Unilateral Deficit	OR = 0.881 *p* = 0.787	OR = 3.064 *p* < 0.001	OR = 0.931 *p* = 0.926	OR = 3.387 *p* < 0.001
Menière	OR = 0.928 *p* = 0.906	OR = 5.211 *p* < 0.001	OR = 3.373 *p* = 0.070	OR = 3.290 *p* = 0.002
Kinetosis	OR = 0.015 *p* < 0.001	OR = 3.278 *p* = 0.124	OR = 91.818 *p* < 0.001	OR = 0.400 *p* = 0.399
Central Vertigo	OR = 0.180 *p* < 0.001	OR = 3.354 *p* < 0.001	OR = 1.413 *p* = 0.598	OR = 2.267 *p* = 0.004
Chronic Instabilities	OR = 0.101 *p* = 0.006	OR = 4.933 *p* = 0.067	OR = 5.347 *p* = 0.096	OR = 12.627 *p* = 0.021

**Table 3:** Logistic regression analysis is used for binary outcome data (e.g., rotatory vertigo) to model associations with each predictor (e.g., BPPV), represented in terms of odds ratios (OR). These ORs are calculated separately for patients with hypothyroidism and for Euthyroid patients.

*Symptoms/Pathologies* *Euthyroidism (0)/Hypothyroidism (1)*		Rotatory Vertigo	Instabilities	Visual Dependency	Walk Disorders
BPPV	0	OR = 10.642	OR = 0.144	OR = 0.032	OR = 0.232
(*n* = 367)		*p* < 0.001	*p* < 0.001	*p* < 0.001	*p* < 0.001
1		OR = 3.983	OR = 0.078	OR = 0.433	OR = 0.160
(*n* = 54)		*p* = 0.214	*p* < 0.001	*p* = 0.385	*p* = 0.006
Unilateral Deficit	0	OR = 1.054	OR = 3.151	OR = 1.085	OR = 3.420
(*n* = 367)		*p* = 0.918	*p* < 0.001	*p* = 0.917	*p* < 0.001
1		OR = 0.130	OR = 2.844	OR = 2.067	OR = 3.143
(*n* = 54)		*p* = 0.112	*p* = 0.192	*p* = 0.697	*p* = 0.152
Menière Disease	0	OR = 0.821	OR = 4.757	OR = 4.312	OR = 2.688
(*n* = 367)		*p* = 0.758	*p* < 0.001	*p* = 0.022	*p* = 0.016
1		OR = 0.579	OR = 9.067	OR = 2.939	OR = 10.000
(*n* = 54)		*p* = 0.645	*p* = 0.028	*p* = 0.747	*p* = 0.020
Kinetosis	0	OR = 0.017	OR = 3.467	OR = 97.50	OR = 0.410
(*n* = 367)		*p* < 0.001	*p* = 0.988	*p* < 0.001	*p* = 0.397
1		OR = NaN	OR = NaN	OR = NaN	OR = NaN
(*n* = 54)		*p* = NaN	*p* = NaN	p= NaN	*p* = NaN
Central Vertigo	0	OR = 0.166	OR = 2.737	OR = 0.477	OR = 2.009
(*n* = 367)		*p* < 0.001	*p* < 0.001	*p* = 0.471	*p* = 0.024
1		OR = 0.182	OR = 9.625	OR = 16.059	OR = 5.500
(*n* = 54)		*p* = 0.197	*p* = 0.003	*p* = 0.024	*p* = 0.020
Chronic Instabilities	0	OR = 0.074	OR = 10.480	OR = 6.712	OR = 28.731
(*n* = 367)		*p* < 0.001	*p* = 0.010	*p* = 0.057	*p* < 0.001
1		OR = 0.146	OR = 0.590	OR = 11.667	OR = 0.640
(*n* = 54)		*p* = 0.843	*p* = 0.457	*p* = 0.890	*p* = 0.475

## Data Availability

No application.
